# Small non-coding RNA biomarkers in sputum for lung cancer diagnosis

**DOI:** 10.1186/s12943-016-0520-8

**Published:** 2016-05-12

**Authors:** Yun Su, Maria A. Guarnera, HongBin Fang, Feng Jiang

**Affiliations:** Department of Surgery, Jiangsu Province Hospital, Nanjing University of Chinese Medicine, 155 Hanzhong Rd., Nanjing, 210029 China; Department of Pathology, The University of Maryland School of Medicine, 10 South Pine Street, MSTF 7th floor, Baltimore, MD 21201-1192 USA; Department of Epidemiology, University of Maryland School of Medicine, Baltimore, MD USA

## Abstract

The early detection of lung cancer can reduce the mortality. However, there is no effective means in clinical settings for noninvasively detecting lung cancer. We previously developed 3 sputum miRNA biomarkers and 2 sputum small nucleolar RNA (snoRNA) biomarkers that can potentially be used for noninvasively diagnosing lung cancer. Here we evaluate the individual and combined applications of the two types of biomarkers in different sets of lung cancer patients and controls. Combined analysis of the miRNAs and snoRNAs has a synergistic effect with 89 % sensitivity and 89 % specificity, and may provide a useful tool for lung cancer early detection.

## Background

Non-small cell lung cancer (NSCLC), primarily caused by cigarette smoking, is the leading cause of cancer-related mortalities [1]. There are two major types of NSCLC: adenocarcinoma (AC) and squamous cell carcinoma (SCC). The early detection of NSCLC may decrease the mortality [1, 2]. However, there is no effective and noninvasive means for the early detection [3]. Sputum is a noninvasively and easily accessible body fluid that contains exfoliated bronchial epithelial cells [4]. Molecular study of sputum could detect the molecular abnormalities in the bronchial airways that reflect those existing in primary lung tumors, and thus provides a noninvasive approach for NSCLC detection [5].

Small non-coding RNAs (ncRNAs) mainly consist of microRNAs (miRNAs) and small nucleolar RNAs (snoRNAs), and play an important role in the pathogenesis of various cancers [6–16]. There is significant interest in the development of the tumor-related ncRNAs as biomarkers for cancer diagnosis [17]. We have identified a panel of three sputum miRNA biomarkers (miRs-21, 31, and 210) with 82.9 % sensitivity and 87.8 % specificity and a panel of two snoRNA sputum biomarkers (snoRDs-66 and 78) with 74.6 % sensitivity and 83.6 % specificity for lung cancer early detection [18–20]. Since lung cancer is a heterogeneous disease featuring field defects in the airway of smokers [21, 22], a single biomarker type can’t achieve the sensitivity and specificity required for clinically diagnosing NSCLC. Because miRNAs and snoRNAs have highly and actively different roles in tumorigenesis, integrating the miRNA and snoRNA-based biomarkers may improve the performance of the biomarkers for NSCLC detection. Here we evaluate the individual and combined applications of the two different types of ncRNAs for the early detection of lung cancer.

## Findings

With a protocol approved by Institutional Review Board of the University of Maryland Medical Center Center, we collected sputum from 316 NSCLC patients and 528 cancer-free smokers. Of the 316 lung cancer patients, 103 were diagnosed with stage I NSCLC. We used the 103 stage I NSCLC patients as cases. From the cancer-free subjects, we randomly selected 117 individuals as control cases. The 103 stage I NSCLC cases and 117 cancer-free smokers were randomly split into a training set (Table 1) and an internal testing set (Table 2).

**Table 1:** Characteristics of lung cancer patients and cancer-free smokers of a training set

	NSCLC cases (*n* = 46)	Controls (*n* = 55)	*P*-value
Age	65.28 (SD 11.27)	67.65 (SD 11.34)	0.35
Sex			0.38
Female	18	22	
Male	28	33	
Race			0.08
White	30	36	
African American	16	19	
Pack-years	44.79 (Range, 5–172)	43.45 (Range, 5–109)	0.38
FEV1/FVC	0.45–0.79	0.43–0.80	0.10
Nodule size (cm)	4.79 (Range, 95.25)	1.29 (Range, 56.76)	<0.01
Stage, all are stage I			
Histological type			
Adenocarcinoma	25		
Squamous cell carcinoma	21		

*Abbreviations*: *NSCLC* non-small cell lung cancer

**Table 2:** Characteristics of lung cancer patients and cancer-free smokers of a testing set

	NSCLC cases (*n* = 57)	Controls (*n* = 62)	*P*-value
Age	64.26 (SD 12.37)	66.69 (SD 10.88)	0.36
Sex			0.39
Female	22	23	
Male	35	39	
Race			0.09
White	37	40	
African American	20	22	
Pack-years	43.89 (Range, 5–170)	42.64 (Range, 5–112)	0.39
FEV1/FVC	0.46–0.78	0.44–0.79	0.09
Nodule size (cm)	4.89 (Range, 96.22)	1.54 (Range, 54.89)	<0.01
Stage, all are stage I			
Histological type			
Adenocarcinoma	31		
Squamous cell carcinoma	26		

*Abbreviations*: *NSCLC* non-small cell lung cancer

**Fig. 1:**
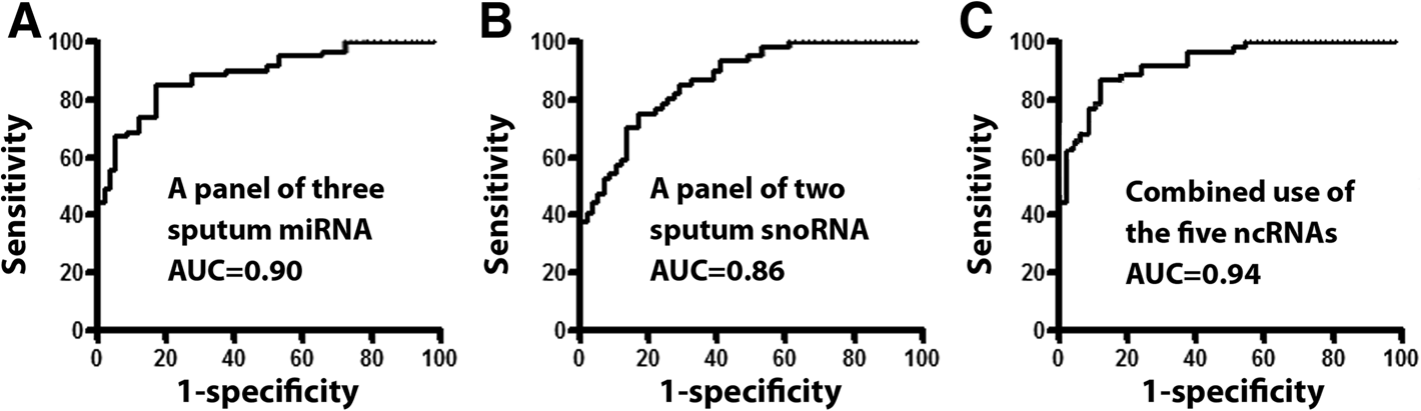
Combined analysis of miRNAs and snoRNAs in sputum has a synergistic effect for lung cancer detection. **a** ROC curve of a panel of three sputum miRNA biomarkers (miRs-21, 31, and 210) shows an AUC of 0.90 for differentiating NSCLC patients from the cancer-free subjects in terms of sensitivity and specificity. **b** a panel of two snoRNA sputum biomarkers (snoRDs-66 and 78) creates an AUC of 0.86 for distinguishing NSCLC patients from the cancer-free subjects. **c** combined study of the three miRNAs and two snoRNAs in sputum yields 0.94 AUC, which is significantly higher than that of any single type of ncRNAs used alone (*P* < 0.05) for lung cancer detection

We determined expressions of the five ncRNAs (miRs-21, 31, and 210, and snoRDs-66 and 78) by quantitative reverse transcriptase PCR (qRT-PCR) in the sputum samples [18, 23–27]. The panel of three miRNAs (miRs-21, 31, and 210) and panel of two snoRNAs (snoRDs-66 and 78) had a receiver operating characteristic (ROC) curve (AUC) value of 0.90 and 0.86, respectively (Fig. 1). Interestingly, combined use of the five ncRNAs produced 0.94 AUC (Fig. 1), being significantly higher than that of the panel of three miRNAs (0.90) or the panel of two snoRNAs (0.86) (*p* < 0.05). Furthermore, combined analysis of the five ncRNAs had higher sensitivity (89.13 % vs. 82.61 % or 73.91 %) and specificity (89.09 % vs. 85.45 % or 83.64 %) compared with the individual panels (All *P* < 0.05). The expression level of the five ncRNAs was associated with smoking history and size of PN of participants (All *P* < 0.05). The expression level of sputum miR-21 was more closely related with AC (*P* < 0.05), whereas miR-210 was associated with SCC (*P* < 0.05). However, overall, the panel of the five ncRNA biomarkers didn’t exhibit special association with a histological type of the NSCLC cases, and the age, gender, ethnicity, and forced expiratory volume 1 (FEV1)/forced vital capacity (FVC) of the participants (All *P* > 0.05). The validation of the ncRNA biomarkers in a testing cohort confirmed that combined study of miRNAs and snoRNAs in sputum had a synergistic effect for the early detection of NSCLC.

## Conclusion

Combined study of the miRNAs and snoRNAs has higher sensitivity and specificity compared with a single type of the ncRNA biomarkers, providing a noninvasive approach for lung cancer early detection. Nevertheless, a prospective project is required for validating the utility.

### Ethical statements

No concern.

## References

[1] Cancer Facts & Figures 2012 (2012). American Cancer Society (ACS). J Consum Health Internet.

[2] Aberle DR, Adams AM, Berg CD, Black WC, Clapp JD, Fagerstrom RM, Gareen IF, Gatsonis C, Marcus PM, Sicks JD (2011). Reduced lung-cancer mortality with low-dose computed tomographic screening. N Engl J Med.

[3] Patz EF, Pinsky P, Gatsonis C, Sicks JD, Kramer BS, Tammemagi MC, Chiles C, Black WC, Aberle DR (2014). Overdiagnosis in low-dose computed tomography screening for lung cancer. JAMA Intern Med.

[4] Saccomanno G, Saunders RP, Archer VE, Auerbach O, Kuschner M, Beckler PA (1965). Cancer of the lung: the cytology of sputum prior to the development of carcinoma. Acta Cytol.

[5] Hubers AJ, Prinsen CF, Sozzi G, Witte BI, Thunnissen E (2013). Molecular sputum analysis for the diagnosis of lung cancer. Br J Cancer.

[6] Croce CM, Calin GA (2005). miRNAs, cancer, and stem cell division. Cell.

[7] Mei Y, Clark D, Mao L (2013). Novel dimensions of piRNAs in cancer. Cancer Lett.

[8] Mannoor K, Liao J, Jiang F (1826). Small nucleolar RNAs in cancer. Biochim Biophys Acta.

[9] Mannoor K, Shen J, Liao J, Liu Z, Jiang F (2014). Small nucleolar RNA signatures of lung tumor-initiating cells. Mol Cancer.

[10] Mei YP, Liao JP, Shen J, Yu L, Liu BL, Liu L, Li RY, Ji L, Dorsey SG, Jiang ZR, Katz RL, Wang JY, Jiang F (2012). Small nucleolar RNA 42 acts as an oncogene in lung tumorigenesis. Oncogene.

[11] Liao J, Yu L, Mei Y, Guarnera M, Shen J, Li R, Liu Z, Jiang F. Small nucleolar RNA signatures as biomarkers for non-small-cell lung cancer. Mol Cancer. 2010;9:198. 10.1186/1476-4598-9-198 PMC2919450 20663213

[12] Dong XY, Rodriguez C, Guo P, Sun X, Talbot JT, Zhou W, Petros J, Li Q, Vessella RL, Kibel AS, Stevens VL, Calle EE, Dong JT. SnoRNA U50 is a candidate tumor-suppressor gene at 6q14.3 with a mutation associated with clinically significant prostate cancer. Hum Mol Genet. 2008;17:1031–42. 10.1093/hmg/ddm375 PMC2923223 18202102

[13] Su H, Xu T, Ganapathy S, Shadfan M, Long M, Huang TH, Thompson I, Yuan ZM. Elevated snoRNA biogenesis is essential in breast cancer. Oncogene. 2014;33:1348–58. 10.1038/onc.2013.89 23542174

[14] Williams GT, Farzaneh F (2012). Are snoRNAs and snoRNA host genes new players in cancer?. Nat Rev Cancer.

[15] Esteller M (2011). Non-coding RNAs in human disease. Nat Rev Genet.

[16] Deng G, Sui G (2013). Noncoding RNA in oncogenesis: a new era of identifying key players. Int J Mol Sci.

[17] Hayes J, Peruzzi PP, Lawler S (2014). MicroRNAs in cancer: biomarkers, functions and therapy. Trends Mol Med.

[18] Shen J, Liao J, Guarnera MA, Fang H, Cai L, Stass SA, Jiang F (2014). Analysis of MicroRNAs in sputum to improve computed tomography for lung cancer diagnosis. J Thorac Oncol.

[19] Xing L, Su J, Guarnera MA, Zhang H, Cai L, Zhou R, Stass SA, Jiang F (2015). Sputum microRNA biomarkers for identifying lung cancer in indeterminate solitary pulmonary nodules. Clin Cancer Res.

[20] Su J, Liao J, Gao L, Shen J, Guarnera MA, Zhan M, Fang H, Stass-Feng Jiang SA, Jiang F (2015). Analysis of small nucleolar RNAs in sputum for lung cancer diagnosis. Oncotarget.

[21] Tang X, Shigematsu H, Bekele BN, Roth JA, Minna JD, Hong WK, Gazdar AF, Wistuba II (2005). EGFR tyrosine kinase domain mutations are detected in histologically normal respiratory epithelium in lung cancer patients. Cancer Res.

[22] Solis LM, Behrens C, Raso MG, Lin HY, Kadara H, Yuan P, Galindo H, Tang X, Lee JJ, Kalhor N, Wistuba II, Moran CA (2012). Histologic patterns and molecular characteristics of lung adenocarcinoma associated with clinical outcome. Cancer.

[23] Anjuman N, Li N, Guarnera M, Stass SA, Jiang F (2013). Evaluation of lung flute in sputum samples for molecular analysis of lung cancer. Clin Transl Med.

[24] Li N, Ma J, Guarnera MA, Fang H, Cai L, Jiang F (2014). Digital PCR quantification of miRNAs in sputum for diagnosis of lung cancer. J Cancer Res Clin Oncol.

[25] Xie Y, Todd NW, Liu Z, Zhan M, Fang H, Peng H, Alattar M, Deepak J, Stass SA, Jiang F. Altered miRNA expression in sputum for diagnosis of non-small cell lung cancer. Lung Cancer. 2010;67:170–6. 10.1016/j.lungcan.2009.04.004 PMC2846426 19446359

[26] Xing L, Todd NW, Yu L, Fang H, Jiang F (2010). Early detection of squamous cell lung cancer in sputum by a panel of microRNA markers. Mod Pathol.

[27] Yu L, Shen J, Mannoor K, Guarnera M, Jiang F (2014). Identification of ENO1 As a Potential Sputum Biomarker for Early-Stage Lung Cancer by Shotgun Proteomics. Clin Lung Cancer.

